# Map-matching algorithm based on the junction decision domain and the hidden Markov model

**DOI:** 10.1371/journal.pone.0216476

**Published:** 2019-05-13

**Authors:** Hui Qi, Xiaoqiang Di, Jinqing Li

**Affiliations:** 1 Jilin Province Key Laboratory of Network and Information Security, Changchun University of Science and Technology, Changchun, Jilin, China; 2 School of Computer Science and Technology, Changchun University of Science and Technology, Changchun, Jilin, China; Zhejiang University, CHINA

## Abstract

Map-matching technology is a key and difficult technology in the development of vehicle navigation systems. Only by correctly identifying the road segment on which the vehicle is traveling can the navigation system make the right decision. At the same time, the complexity of the road network structure and a variety of error factors have introduced great challenges to map matching and have attracted the attention of many researchers as well. This paper analyzes various map-matching algorithms, determines that the key to the matching performance is the junction matching, performs an in-depth study on the junction-matching problem, and puts forward the junction decision domain model. The model mainly involves information regarding the width of the road segment, the angle between two road segments, the accuracy of GPS and the accuracy of the road network. In this paper, we use this model to improve the map-matching algorithm based on a hidden Markov model (HMM). The experimental results show that the improved matching algorithm can effectively reduce the error rate of junction matching and improve the matching performance of a navigation system.

## 1 Introduction

Map matching is the basis of intelligent transportation applications such as vehicle tracking, traffic flow monitoring, travel time forecasting, and route planning. Thus, map matching occupies an important place in dynamic onboard navigation systems. Only after determining the road on which the vehicle is traveling can the navigation system provide the driver with correct navigation information.

In brief, the issue of map matching can be described as determining how to position a random GPS point or a set of such points to correspond to the most likely road segment (line segment). This issue has now been specifically studied in much of the literature. Earlier studies mainly focused on geometrical analysis or geometrical analysis plus road network analysis. However, the inherent limitations of geometrical analysis imposed restrictions on the improvement in matching accuracy. Thus, the studies on nongeometrical analysis methods, such as those based on probability theory, fuzzy logic theory and evidence theory, were initiated and yielded good results. Despite the availability of higher-level matching methods, scenes such as intersections and parallel roads were still hard to match, incurring a high mismatch rate [1–4]. To solve the problem of poor matching in a complex road network, the references [1, 5, 6] proposed that the road width can be introduced into the matching algorithm. This paper draws on the idea of the road width, proposes the junction decision domain model, and uses this model to improve the widely implemented HMM-based matching algorithm in order to improve the junction matching performance.

The main contributions of this article are the following: 1. The construction method of the junction decision domain is proposed, and information regarding the width of the road segment, the accuracy of the GPS, the accuracy of the road network and the angle between the road segments is used to obtain more accurate intersection information. 2. The junction decision domain is introduced in the HMM-based matching algorithm, and different matching strategies inside and outside the intersection are used to obtain better junction matching performance. 3. The proposed algorithm is compared with the traditional HMM-based matching algorithm using a public data set and the data set of this paper. Experiments show that the reasonable setting of the junction decision domain is helpful for improving the matching performance, especially the junction matching performance.

In the following sections, we first briefly introduce the research on the map-matching algorithm, especially the algorithm based on HMM; then, we analyze the problem of junction matching and propose the junction decision domain model and corresponding algorithm to solve the problem; and finally, we conduct some field tests to verify the performance of the model and the algorithm.

## 2 Related work

In the literature [7, 8], some matching algorithms that are based on geometric analysis methods have been introduced. For example, GPS points are positioned to the nearest road network nodes (point-to-point matching) or the nearest road segments (point-to-curve matching). Alternatively, a GPS trajectory is matched to the nearest continuous road segments (curve-to-curve matching). In addition to pure geometrical analysis, road network analysis is used in some of the algorithms in [7, 8]. After the road network analysis, an algorithm can be divided into two parts, namely, initial matching and subsequent matching. Initial matching determines the road segment to be matched through geometrical analysis, while subsequent matching selects candidate segments according to the previous matching result through both geometrical analysis and road network analysis. Candidate road segments are a set of segments consisting of the previously matched segment and the segments directly connected to it. Once candidate segments are identified, the best segment to be matched can be chosen through geometrical analysis. The test result of [8] shows that there is not much difference in the matching performance between pure geometrical analysis and geometrical analysis plus road network analysis, both with a matching accuracy of 66%-86% and a high mismatch rate at intersections—an important reason for low matching accuracy. The algorithms proposed in the above references are usually used for real-time matching, where the sampling rate of GPS signals is high and the algorithms can immediately address map matching and output the matching results as soon as GPS signals are received. The opposite of realtime matching is non-realtime matching. At this time, the sampling rate is relatively low, and the matching algorithm does not output a matching result for each GPS signal but instead outputs the result after receiving a certain number of signals. In [9], a non-realtime matching algorithm was proposed. This algorithm is different from curve-to-curve matching and functions by using the Fréchet distance to calculate the distance between the vehicle trajectory curve and road curve. However, since the essence of this algorithm is also geometric analysis, it is impossible to completely remove the defects of the geometric analysis method.

Considering the sensitivity of geometrical analysis to the geometric contour, the measurement noise and the sampling rate, many scholars have introduced some nongeometric methods in their studies. In [10, 11], MHT is used to improve the selection process of candidate road segments with a different approach from that in [7, 8]. Instead of assuming that the previous matched road segment is correct, the MHT method bases the current road network analysis on all the previous candidate road segments to establish a new set of candidate segments. MHT is helpful in avoiding continuous mismatches (the *k*th mismatch leads to the *k* + 1th one), thus playing a certain role in improving the matching performance at intersections and on parallel roads. In [12], a matching algorithm based on fuzzy logic theory was proposed and divided into initial matching and subsequent matching. This algorithm at first calculates the input values of the navigation system through geometrical analysis and road network topology analysis, then converts the input values into fuzzy memberships, and finally finishes matching by applying the fuzzy rules to fuzzy memberships. The most important aspect in this algorithm is determining the fuzzy memberships and fuzzy rules involving many parameters, whose values are determined by experience and by sample training based on establishing a neural network. One drawback of this algorithm is that the initial matching requires a longer time and will be restarted if the subsequent matching is wrong. In [5], a matching algorithm based on interval analysis and evidence theory was proposed. The approach takes the road network topology and road width into account, models the road width and GPS error through interval analysis, calculates the mass function, combines two types of evidence (one is predictive evidence derived from road network topology analysis; the other is observable evidence derived from interval analysis) according to the combination rule of evidence theories, and finally determines the road segment to be matched in light of the combined evidence. The algorithm in [5] shares the same idea as MHT in the road network topology analysis and achieves a matching accuracy approximately 11% higher than that of the geometrical analysis method in [7, 8]. In recent years, the HMM-based matching algorithm has received attention from many researchers [6, 13–21]. This algorithm calculates the observation probability through geometrical analysis and the state transition probability through road network topology analysis and geometrical analysis, and finally chooses the road segment to be matched through the Viterbi algorithm or through its improved version. The strongest point of the algorithm is its insensitivity to abnormal data. In addition, it behaves well at a lower sampling rate. As reported in [6], the matching accuracy can reach approximately 85% during a 50-100 s sampling interval and even as high as 95% at a high sampling rate.

To summarize, geometrical analysis is the basis of any map-matching algorithm. Even in those advanced algorithms that employ nongeometrical analysis, the values derived from geometrical analysis are also important inputs. Road network topology analysis—especially that based on MHT—is a key approach to improve the map matching. At present, both the matching algorithms based on interval analysis and evidence theory and those that are HMM-based are characterized by MHT in the road network topology analysis. Road network accuracy and road width have become two important reference factors in any matching algorithm [3]. The HMM-based algorithm, which is insensitive to measurement noise and sampling rate, is fit for both realtime and non-realtime matching. In terms of non-realtime matching, this algorithm can correct historical errors and is less complicated to calculate than the algorithm based on Fréchet distance. The current studies on the HMM-based algorithm focus mainly on non-realtime matching, featuring a low sampling rate [6, 13–21]. This algorithm can be applied to the server of a dynamic navigation system to monitor vehicle routes or traffic flows. However, the client of a navigation system needs a realtime matching algorithm appropriate for a high sampling rate. Although the HMM-based matching algorithm adapts to this requirement, the matching algorithm cannot improve the accuracy by correcting historical errors unlike the non-realtime matching. For example, at the time *k*, the matching algorithm yields the wrong result. For the non-realtime matching, this error can be corrected by using the data collected later, and the corrected result can be accepted. However, for the realtime matching, this error, even when corrected, cannot be treated as a correct result. During the realtime matching with a high sampling rate, special attention must be paid to intersection environments that easily cause the mismatch because junction matching is the key constraint on algorithm performance [3]. The algorithm studied in this paper is an HMM-based algorithm that introduces the element of the intersection into the matching result and proposes a key concept—junction decision domain. This concept involves the model of the junction decision domain and the algorithm in that domain. Next, the HMM-based algorithm that is improved in this paper will be introduced.

## 3 HMM-based matching algorithm

### 3.1 Introduction

The hidden Markov model (HMM) has been applied in many fields. The more common applications include speech recognition and part-of-speech tagging. HMM is briefly introduced here. This model is based on a Markov chain, which means that with the current knowledge or information given, only the current state can be used for the future state prediction, while the previous (before now) historical state is irrelevant to the future (after now) state prediction. The nature of a Markov chain can be expressed by the following equation:

$$\begin{array}{c} P\left(X_{n+1}=x|X_{0},X_{1},X_{2},\ldots ,X_{n}\right)=P\left(X_{n+1}|X_{n}\right) \end{array}$$

that is, with the state *X*_*n*_ given, *X*_*n*+1_ is only related to *X*_*n*_ but is irrelevant to the previous n-1 states.

**Fig 1:**
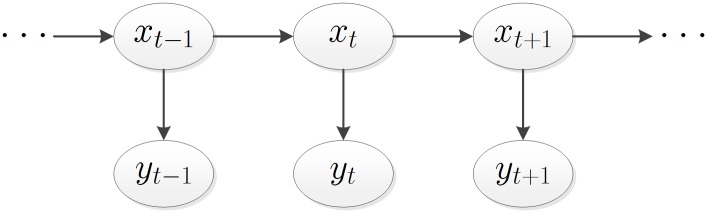
Evolution of HMM model.

In the so-called HMM model, the above Markov states are invisible, while the variables (observable phenomena) influenced by those states are visible. For example, in Fig 1, *x*_*t*−1_, *x*_*t*_ and *x*_*t*+1_ are fit for the Markov process but unobservable, whereas the variables *y*_*t*−1_, *y*_*t*_ and *y*_*t*+1_ are observable but influenced by their own state variables.

From the above description and Fig 1, five basic elements of HMM can be derived:

- Number of state variables for HMM: *N*;
- Number of observable variables for HMM: *M*;
- State transition matrix (the connection between *x*_*t*_ in Fig 1): *A* = [*a*_*ij*_]_*N*×*N*_, where *a*_*ij*_ = *P*(*x*_*t*+1_ = *j*|*x*_*t*_ = *i*), 1 ≤ *i*, *j* ≤ *N*;
- Observation probability matrix (the connection between *x*_*t*_ and *y*_*t*_ in Fig 1): *B* = [*b*_*jk*_]_*N*×*M*_, where *b*_*jk*_ = *P*(*y*_*t*_ = *k*|*x*_*t*_ = *j*), 1 ≤ *j* ≤ *N*, 1 ≤ *k* ≤ *M*;
- Initial state probability vector: $\vec{\pi }=\left(\pi_{i}\right)$, where *π*_*i*_ = *P*(*x*_*t*_ = *i*), 1 ≤ *i* ≤ *N*.

If HMM is applied to map matching, the state variable will be the road segment where the vehicle is actually located, and the observable variable is the output of the GPS sensor. Among the five basic elements, the set of state variables is determined in real time through road network analysis, so the number of state variables (N) is a variable. On the other hand, observable variables are the outputs of the GPS sensor at a moment, so the number of observable variables (M) is 1.

The calculation method of the observation probability matrix is usually determined by the Gaussian distribution of the distances from GPS points to road segments. In [13–15, 22], this probability is calculated with the following equation:

$$\begin{array}{c} P\left(p_{t}|r_{i}\right)=\frac{1}{\sqrt{2\pi }\sigma }exp(-0.5{\left(\frac{\left\|p_{t}-z_{t,i}\right\|}{\sigma }\right)}^{2}) \end{array}$$ (1)

where *p*_*t*_ is the position of the GPS at the time *t*; *r*_*i*_ is the candidate road segment; *z*_*t*,*i*_ is the projection point of the point *p*_*t*_ on the road segment *r*_*i*_; and *σ* is GPS accuracy. In [6], Eq (1) is improved through the addition of road width:

$$\begin{array}{c} \begin{array}{cc} P(p_{t}|r_{i})= & \frac{1}{2w}\int_{-w}^{w}\frac{1}{\sqrt{2\pi }\sigma }\\ & exp(-0.5{\left(\frac{x-\|p_{t}-z_{t,i}\|}{\sigma }\right)}^{2})dx \end{array} \end{array}$$ (2)

where *w* is half of the road width. One significant improvement after the addition of road width is that *P*(*p*_*i*_|*r*_*i*_) cannot depend completely on the distances from GPS points to candidate road segments but rather on both distances and road widths. In Eq (1), the longer the distance from a GPS point to a road segment is, the smaller the possibility that the GPS point is on that segment. However, in Eq (2), a larger road width does not necessarily mean that there is a much smaller possibility that the GPS point is on that road segment. Eq (2) is more practical than Eq (1), but it needs a known road width (which is not definitely available in all the road network data). If *w* is set as a constant, the road width will no longer function so that Eq (2) will revert to Eq (1). In addition, there is a coefficient $\frac{1}{2w}$ in Eq (2) that does not yield a probability in the calculated results of the equation. This coefficient can be removed to obtain a true probability of observation:

$$\begin{array}{c} \begin{array}{cc} P(p_{t}|r_{i})= & \int_{-w}^{w}\frac{1}{\sqrt{2\pi }\sigma }\\ & exp(-0.5{\left(\frac{x-\|p_{t}-z_{t,i}\|}{\sigma }\right)}^{2})dx \end{array} \end{array}$$ (3)

The state transition probability can be derived from road network analysis or from the calculation combining the road network analysis with the GPS output. In [13], this probability is calculated with the following equation:

$$\begin{array}{c} \begin{array}{c} P(r_{t+1,j}|r_{t,i})=\beta \times \\ exp(-\beta \times |\left\|p_{t}-p_{t+1}\right\|-\left\|z_{t,i}-z_{t+1,j}\right\||) \end{array} \end{array}$$ (4)

where *P*(*r*_*t*+1,*j*_|*r*_*t*,*i*_) is the transition probability that the vehicle moves from the road segment *r*_*i*_ at the time *t* to the segment *r*_*j*_ at the time *t* + 1; *β* is an undetermined coefficient, derived from a large amount of sample training. ‖*p*_*t*_ − *p*_*t*+1_‖ is the distance between the GPS point at the time *t* and the point at the time *t* + 1; ‖*z*_*t*,*i*_ − *z*_*t*+1,*j*_‖ is the distance between the projection point on *r*_*i*_ at the time *t* and the point on *r*_*j*_ at the time *t* + 1, which is the distance of the route. Eq (4) can be simplified or expanded. For example, in [14], it is simplified to:

$$\begin{array}{c} P\left(r_{t+1,j}|r_{t,i}\right)\propto exp(-\beta \times |\|z_{t,i}-z_{t+1,j}\||) \end{array}$$ (5)

The reason for this simplification is that, in the author’s view, Eq (4) relates the state transition probability to observable variables and thus violates the HMM assumption. However, in reality, *z*_*t*+1,*j*_ is also calculated by using observable variables, so Eq (5) is indirectly related to those variables as well. In [16] and [23], a method relying completely on road network analysis to determine the state transition probability (whose calculation is independent of observable variables,) was proposed. However, this method may result in the same transition probability for all the candidate roads so that the transition probability will make no contribution to the matching result. In [6], Eq (4) is expanded to include the momentum change. After being expanded, this method is very suitable for non-realtime matching that features a low sampling rate because during the realtime matching, it tends to provide a larger transition probability for state transitions of lanes with straight movement (where the momentum change is smaller) at any intersection. Therefore, in this paper, Eq (4) will be used to calculate the transition probability.

The initial state probability *P*(*p*_0_|*r*_*i*_) is the observation probability at the initial time and can be derived from Eq (1).

Once the five elements of HMM and a series of observable variables (GPS data) are determined, the Viterbi algorithm can be used to solve the most probable state sequence (the matched road segments). Since the time complexity of the Viterbi algorithm is Θ(*nm*^2^) and the space complexity is Θ(*nm*^2^) (where n is the number of observable variables and m is the number of state variables), the Viterbi algorithm will occupy a large amount of computing resources and storage resources when n and m are large. To improve the algorithm performance, the online Viterbi algorithm can be used [24, 25]. In [6], the online Viterbi algorithm was improved through the introduction of a bounded variable sliding window (BVSW) to further reduce the need for computing and storage resources.

### 3.2 Matching algorithm based on junction decision domain

#### 3.2.1 Brief description of matching algorithm

**Fig 2:**
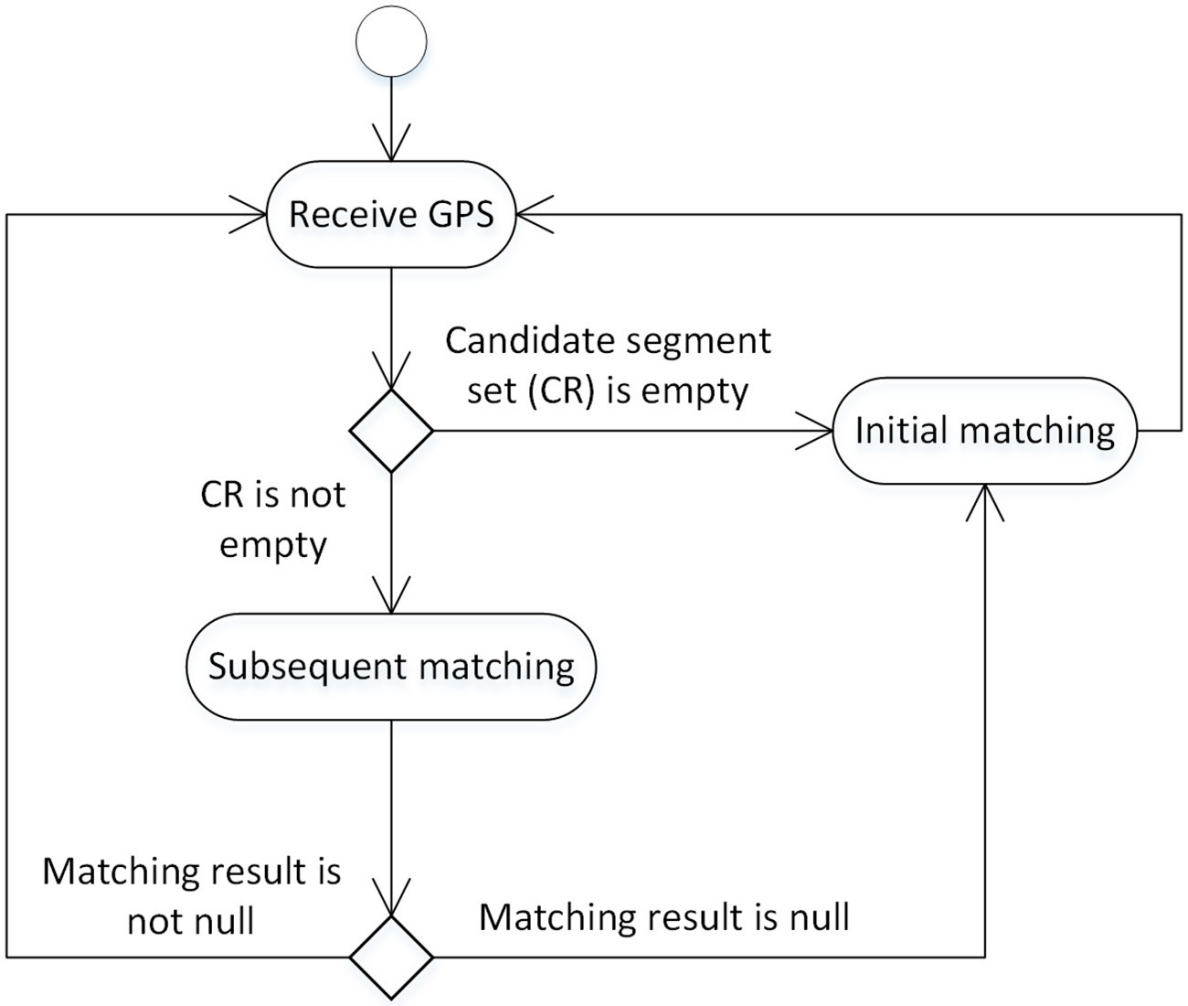
HMM-based matching algorithm without the junction decision domain.

The general flow of the HMM-based real-time matching algorithm that does not implement the junction decision domain is shown in Fig 2. (The junction decision domain is a circular area centered on the intersection. The next section will describe in detail how to calculate the radius of the area.)

Regardless of the initial matching (InitialMatching) or the subsequent matching (SubsequentMatching), the candidate road segments are first determined according to the road network analysis result, and the observation probability of the candidate matching points is calculated. For the initial matching, the observation probability of the candidate matching point is directly taken as its local probability; then, the candidate matching point with the largest local probability is selected as the final matching result. For subsequent matching, it is also necessary to calculate the transition probability of the candidate matching point and multiply the transition probability, the observed probability and the local probability of the last candidate matching point to obtain the local probability of the current candidate matching point; then, the candidate matching point with the largest local probability is selected as the final result. Since the real-time matching does not use the junction decision domain, there is no need to calculate the backward pointer (the backward pointer is used to calculate or correct the last implied state), that is, the previous matching result is not corrected according to the current matching result.

**Fig 3:**
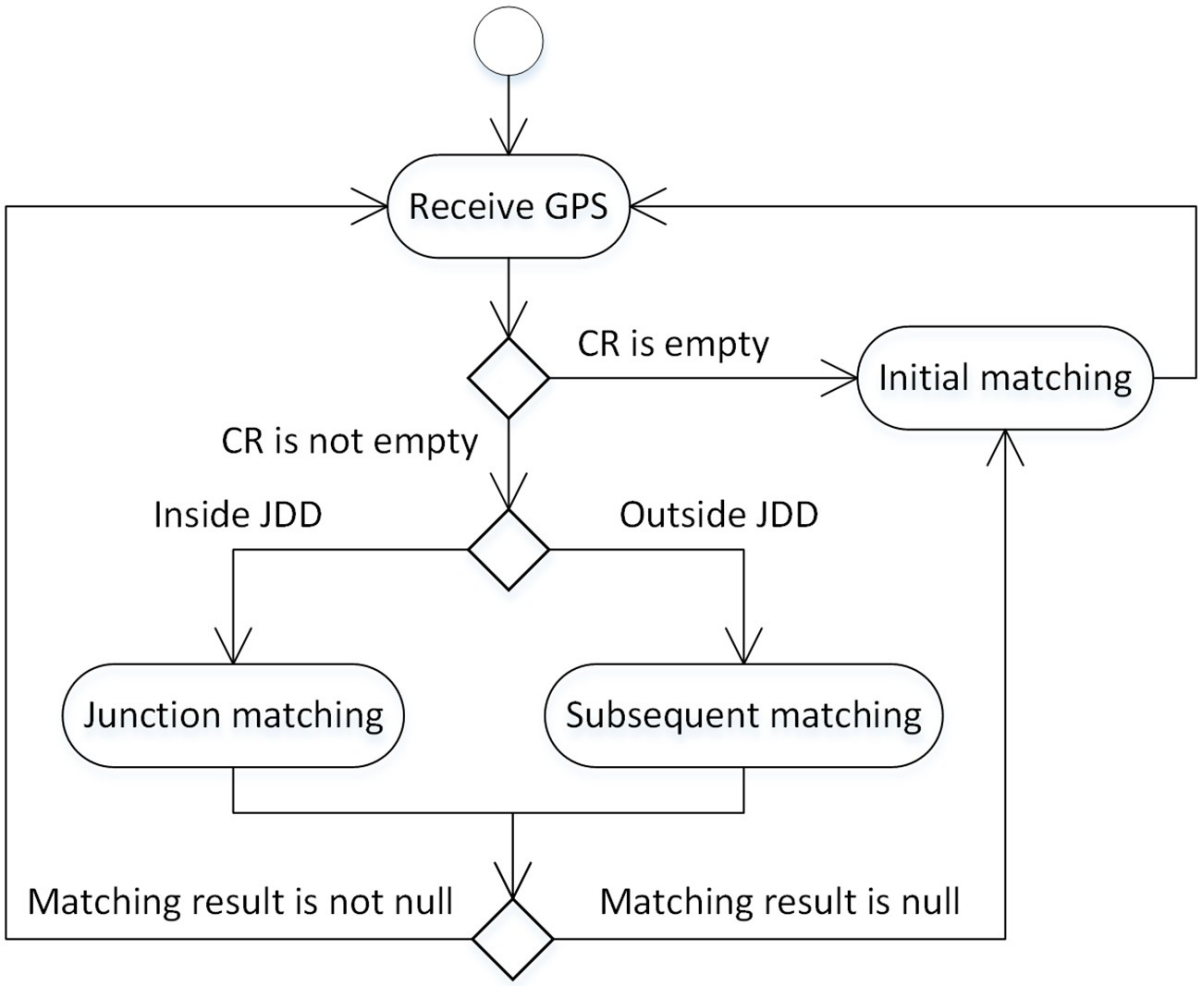
HMM-based matching algorithm using the junction decision domain.

The general flow of the matching algorithm that implements the junction decision domain is shown in Fig 3. Different from the flow of Fig 2, the algorithm introduces the judgment of the junction decision domain and the junction matching (JunctionMatching) when the candidate road segment set (CR) is not empty. When the vehicle is outside the junction decision domain (outside JDD), the matching algorithm performs subsequent matching; otherwise, the junction matching is performed.

The junction matching is an extension to the subsequent matching. The backward pointer needs to be calculated, and the candidate matching point with the largest local probability cannot be directly used as the final result. Instead, an evaluation is made regarding whether the candidate matching point and the previous candidate matching point are on the same road. If the road is the same, it can be used as the final result. Otherwise, it indicates that the vehicle has passed the intersection but has not left the decision domain. At this point, the intersection point should be used as the matching result.

The algorithm also modifies the subsequent matching. If the vehicle is in the junction decision domain at the last moment, it needs to calculate the backward pointer and find all the matching points in the junction decision domain along the backward pointer of the current matching point. If the vehicle is not in the junction decision domain at the last moment, the unmodified subsequent matching is performed, that is, the candidate matching point with the largest local probability is selected as the matching result without calculating the backward pointer. The initial matching, junction matching, and modified subsequent matching algorithms are detailed in Section 3.2.4.

The core of the algorithm in this paper is the junction decision domain. The road network structure in this domain is more complicated. When the vehicle is traveling in the domain, the most likely candidate matching point cannot be directly used as a matching result, but it should be considered whether the vehicle passes the intersection. If the vehicle is still driving on the road before the intersection, the most likely candidate match point is output; otherwise, the intersection point is used as a match until the vehicle leaves the decision domain. At this time, the backward direction pointer is used to completely describe the driving situation of the vehicle in the decision domain, thereby improving the matching performance near the intersection.

It is very important to determine the scope of the junction decision domain reasonably. There are many factors involved. It is necessary to comprehensively consider the width of the road segment, the angle between the road segments at the intersection, the GPS accuracy and the road network accuracy. In addition to comparing the algorithm of this paper with the algorithm without the junction decision domain, the subsequent experiments in this paper will compare the matching algorithms using different decision domains.

#### 3.2.2 Issue of junction matching

**Fig 4:**
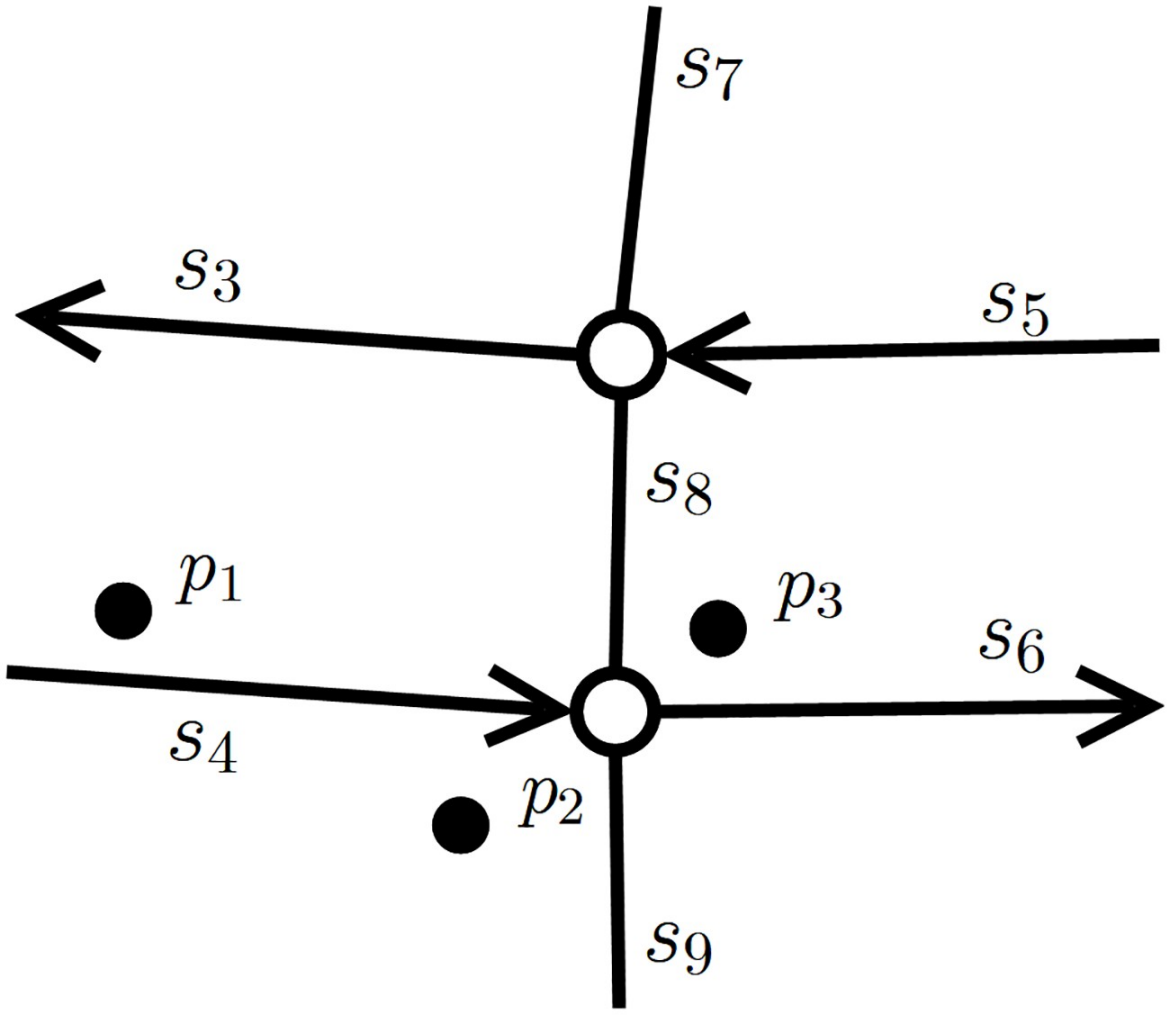
Current vehicle positions and the nearby road network.

This section and the next section will detail the origin of the junction problem and how to calculate the junction decision domain. In the matching process, an error is most likely to occur when the vehicle is driven to an area near a junction. As shown in Fig 4, the points *p*_1_, *p*_2_ and *p*_3_ are the GPS positions at three sequential moments. Among them, the most error-prone points are *p*_2_ and *p*_3_. However, junction matching is the most important. A junction mismatch will have a certain influence on the subsequent operation of the navigation system. Suppose the vehicle is actually moving on the road chain <*s*_4_, *s*_6_> but the navigation system matches the point *p*_3_ to the road segment *s*_8_. Since the segments *s*_8_ and *s*_6_ are not on the same road chain, the navigation system will give an incorrect prompt.

**Fig 5:**
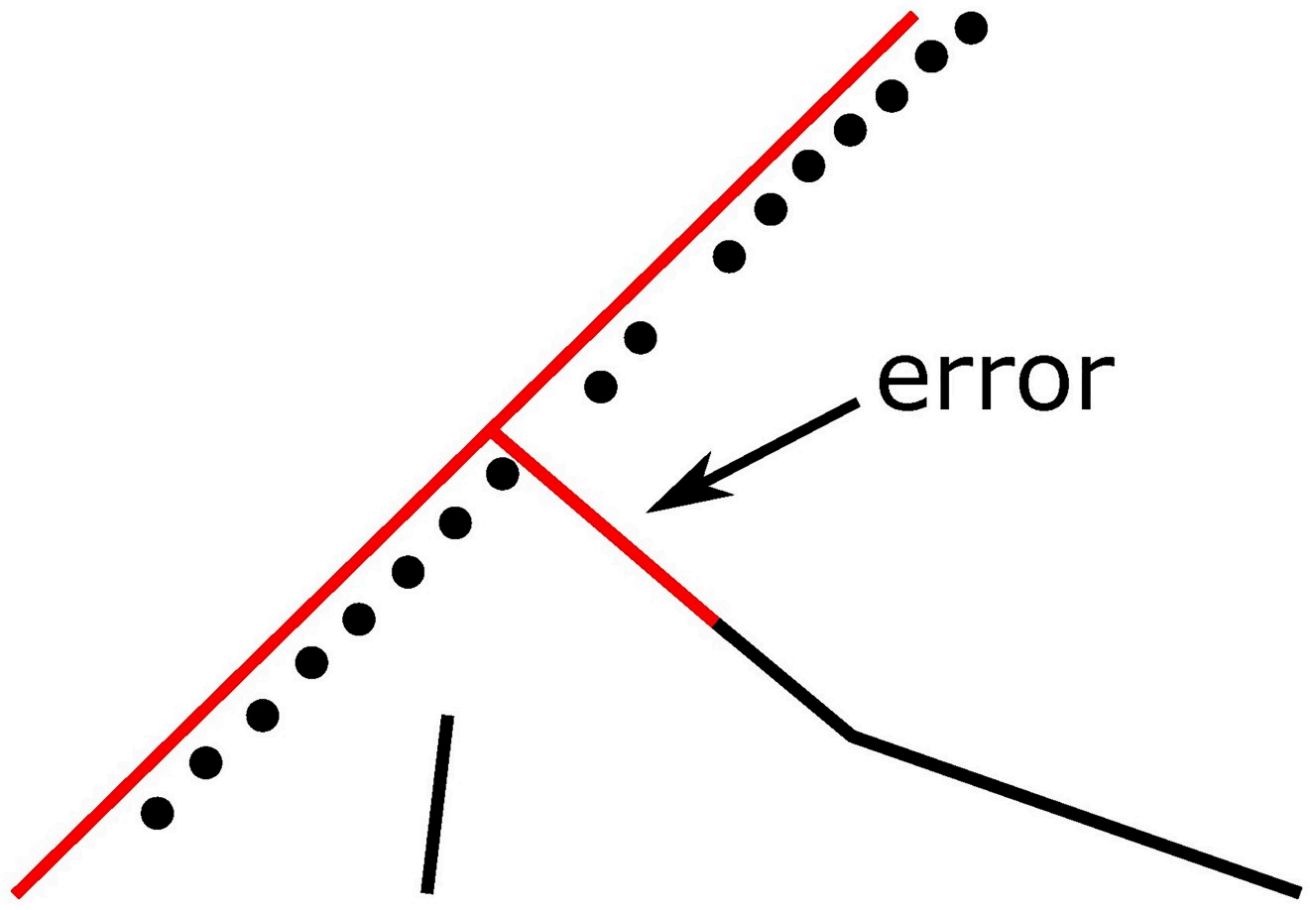
Part of the test chart of a normal junction.

**Fig 6:**
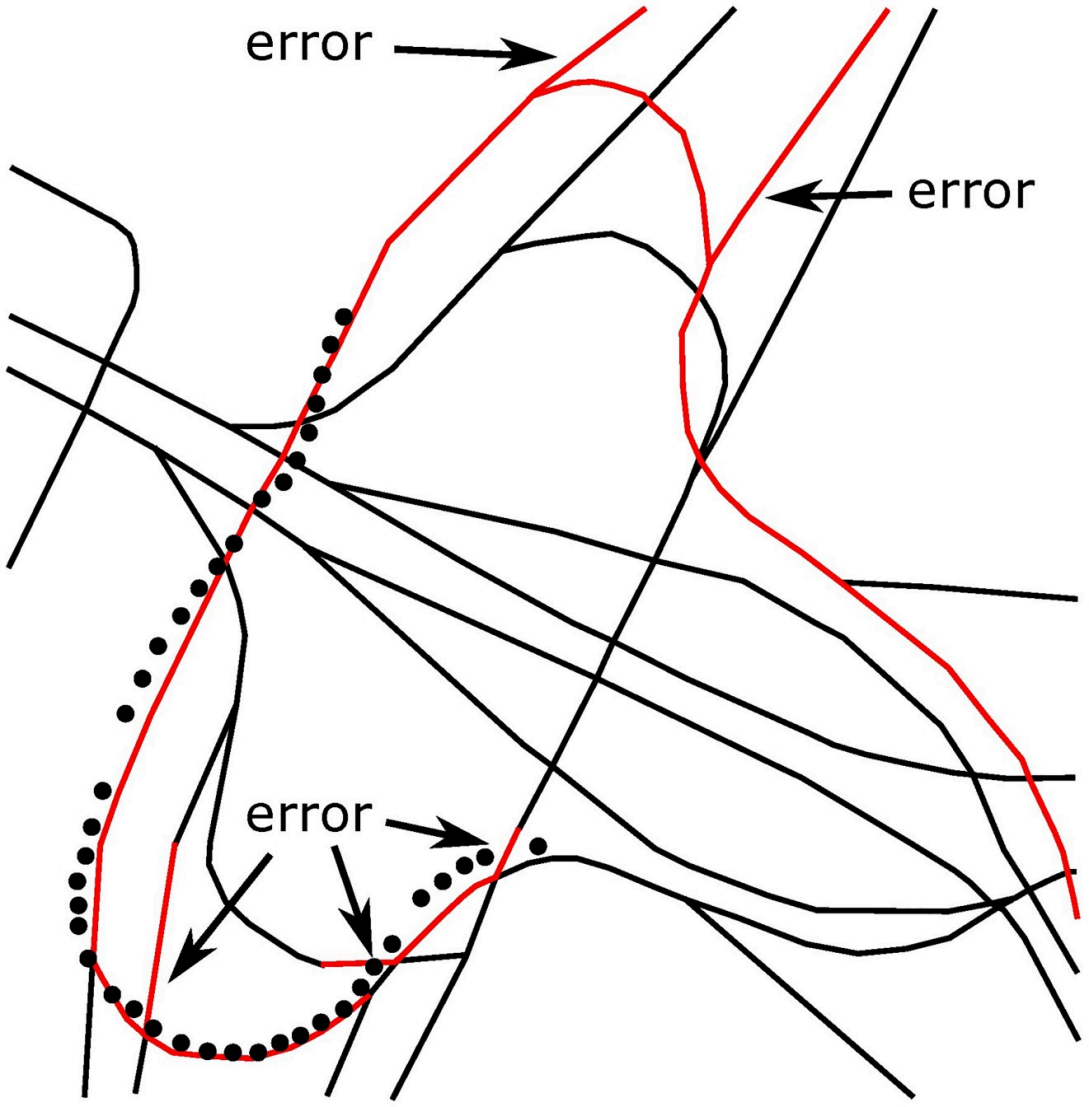
Part of the test chart of an overpass.

In this paper, the matching performance of the HMM-based algorithm at junctions has been specifically tested for a total distance of approximately 30 km by using the testing algorithm proposed in [13] and [14] and the GPS data collected in a real road test. The mismatches caused by the algorithm in [13] were found at 20 junctions among all 145 junctions, contributing to an error rate of 13.8%. Thus, it can be seen that if junctions have not been specifically addressed, the reliability of the navigation system will be seriously affected. In the test, this paper analyzes the test result by coloring the road network structure, as partially shown in Figs 5 and 6, where red roads are the matched ones calculated by the navigation system and the black points are the positions obtained from a GPS sensor at a certain time. Mismatches can be seen clearly in the two figures, especially in Fig 6. Since the overpass has a very complex road network and many junctions, five mismatches have occurred in the navigation system.

#### 3.2.3 Model of the junction decision domain

This paper improves the computing method of the junction decision domain proposed in [1], providing a more reasonable method. It is required in [1] that all the widths of the roads connected to a junction are known, and the value of the decision domain is 1/2 of the previous road width. However, this assumption is too idealistic. First, there is often no road width information in the road network data. Similarly, in the road network data of this paper, no road segments have any width information. Therefore, it is necessary to devise a method of inferring the width information of the road segment, such as inferring by the road grade information. Moreover, it is unreasonable to set the decision domain as 1/2 of the previous road width unless every road connected to a junction is at an angle of 90° and both the road network data and the GPS data are without errors. However, in practical applications, these two conditions are very hard to satisfy, as either the road network data or the GPS data are not completely accurate [26].

**Fig 7:**
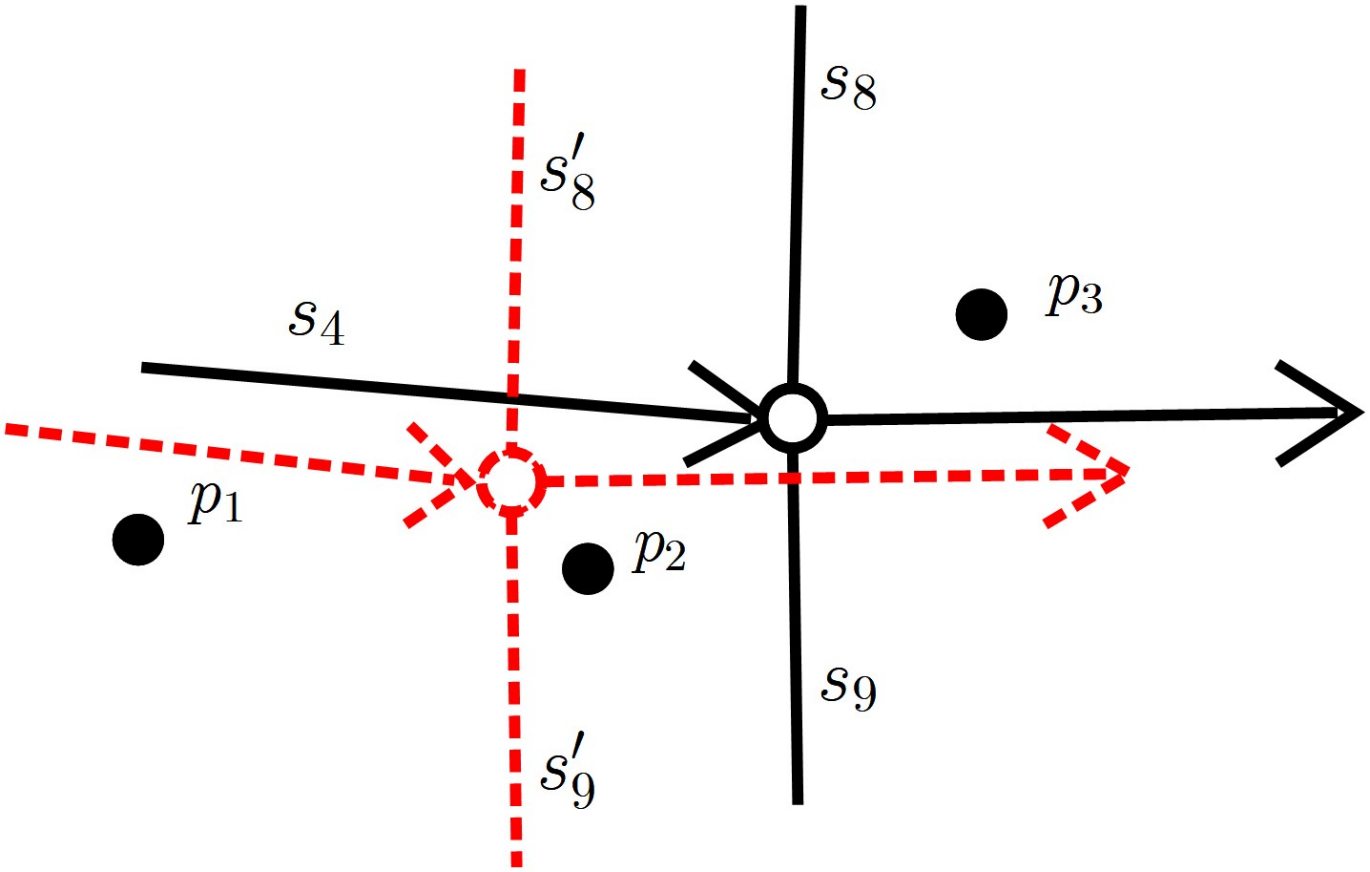
Translation error of the road network structure.

For example, consider the scene in Fig 7. The actual position of a road segment is expressed by a solid line, whereas its position in the road network data is expressed by a dotted line. In Fig 7, translation errors are shown. The distance between the roads *s*_8_ and *s*_8_′ is the error in *s*_8_, or it approximates the error in *s*_9_ or in junction c. *p*_1_, *p*_2_ and *p*_3_ are the GPS positions of the vehicle at *t*_1_, *t*_2_ and *t*_3_, respectively. It can be seen that when the vehicle is driven to *p*_2_, its position is judged as being on the road segment *s*_4_ by the real road matching but possibly on the segment *s*_9_′ by the road network matching. The main reasons for this mistake lie in the error in the road network data, in addition to the GPS error. It is assumed that *e*(*t*) is the GPS error at the time *t*, *e*′(*c*) is the error of junction c, and *l*(*c*) is the width of junction c; then, the decision domain for the dashed junction c in Fig 7 at the time *t* is defined as:

$$\begin{array}{c} r\left(c,t\right)=e\left(t\right)+e^{\prime }\left(c\right)+l\left(c\right) \end{array}$$ (6)

where *r*(*c*, *t*) is the decision domain radius of junction c at the time *t*.

Eq (6) is a function related to the junction and time. In practice, the GPS error at t can be expressed by the GPS precision *σ*, and the error at the junction c can be expressed by the road network precision *σ*′. Therefore, Eq (6) can be rewritten as:

$$\begin{array}{c} r\left(c\right)=\sigma +\sigma^{\prime }+l\left(c\right) \end{array}$$ (7)

**Fig 8:**
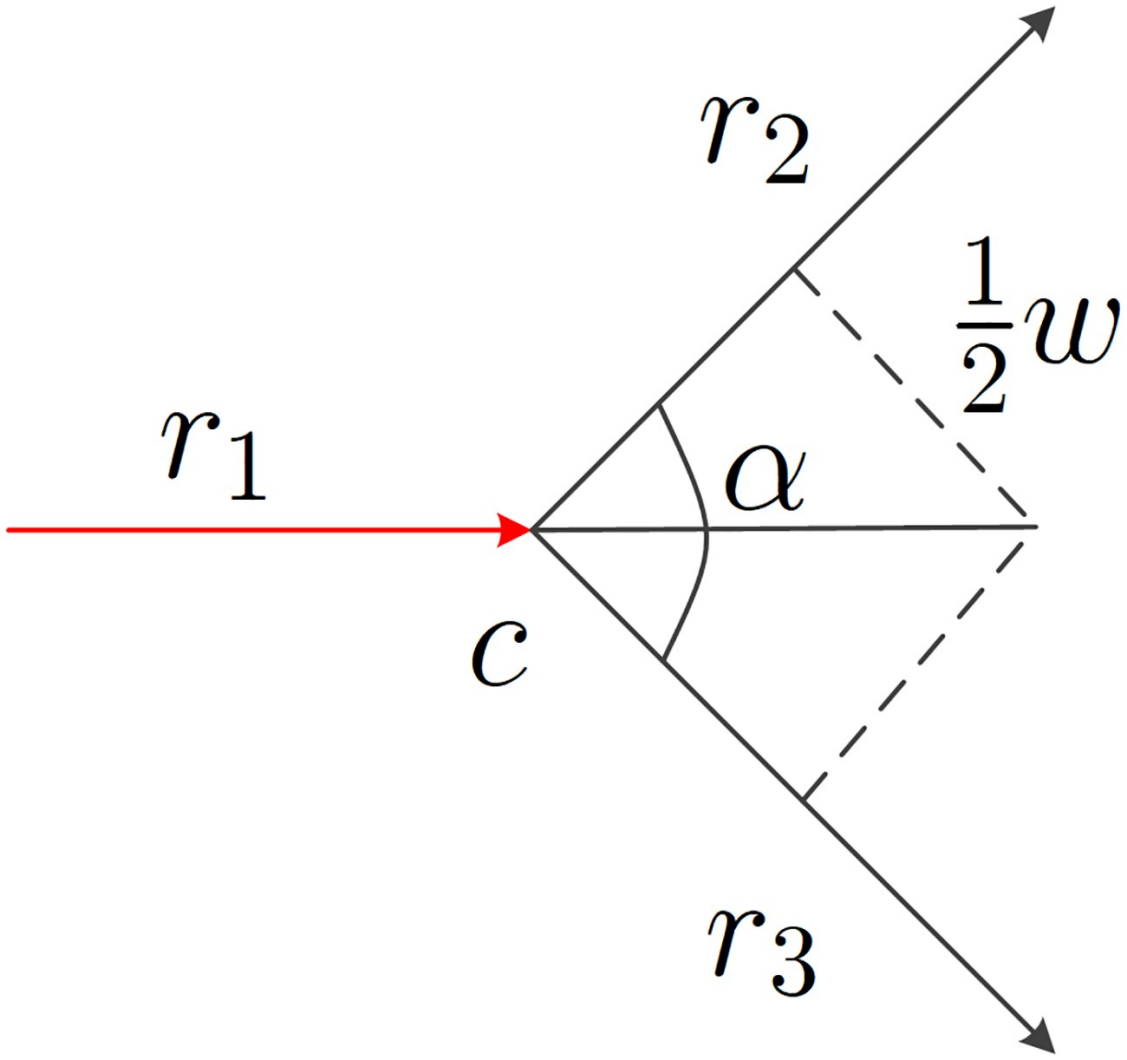
Calculation of the intersection width.

Eq (7) is the junction decision domain model used in this paper. The model is time independent and only related to the junction. Therefore, it is only necessary to find the value of the component *l*(*c*) in the equation to determine the decision domain of the junction c. The value of *l*(*c*) is related to the angle between the road segments at the junction and their widths. As shown in Fig 8, assuming that the vehicle enters the junction from the road segment *r*_1_ and then leaves it from the segment *r*_2_ or *r*_3_ (the width of the road segments *r*_2_ and *r*_3_ is the same; both are *w*, and the angle between the segments *r*_2_ and *r*_3_ is *α*), the width of the junction c can be calculated by:

$$\begin{array}{c} l\left(c\right)=\frac{\frac{1}{2}w}{sin(\frac{1}{2}\alpha )} \end{array}$$ (8)

If the road segment *r*_2_ is not as wide as the segment *r*_3_, the width of junction c cannot be determined according to Eq (8). In this case, the road segment *r*_2_ can be translated downward for a distance of $\frac{1}{2}w_{r_{2}}$ to obtain a new line *r*_2_′ (suppose the width of *r*_2_ is $w_{r_{2}}$), while the segment *r*_3_ is translated upward for a distance of $\frac{1}{2}w_{r_{3}}$ to obtain a new line *r*_3_′. Then, the intersection *c*′ between the lines *r*_2_′ and *r*_3_′ can be solved, and the distance between the junctions c and *c*′ is calculated to determine the width of junction c (*l*(*c*)). Of course, when calculating the junction width, this method is more complicated than Eq (8). If the width difference between the roads *r*_2_ and *r*_3_ is not large, for example $|w_{r_{2}}-w_{r_{3}}|\leq 2\;m$ or $|\frac{1}{2}w_{r_{2}}-\frac{1}{2}w_{r_{3}}|\leq 1\;m$, Eq (8) can be rewritten as:

$$\begin{array}{c} l\left(c\right)=\frac{\frac{1}{2}max(w_{r_{2}},w_{r_{3}})}{sin(\frac{1}{2}\alpha )} \end{array}$$ (9)

#### 3.2.4 Matching algorithm details

This section will describe in detail the three key parts of the algorithm: the initial matching, the subsequent matching and the junction matching. To better describe the algorithm, we need to define the following collections and functions:

*G* Road network with road width information. If the road segment has no width information, the width can be inferred by the road grade.
*MR* The set of matching results, in which each element is a 4-tuple (*r*_*t*_, *z*_*t*_, *δ*_*t*_, *bp*_*t*_), where *r*_*t*_ is the candidate road segment at the time *t*, *z*_*t*_ is the point closest to the GPS point *p*_*t*_ at the time *t* on the road segment *r*_*t*_, *δ*_*t*_ is the local probability, and *bp*_*t*_ is the backward pointer.
**setMR**(*MR*, *t*, *mr*) Modify the matching result at the time *t* in *MR* to *mr*.
**getMR**(*MR*, *t*) Get the matching result at the time *t* in *MR*.
*CR* The set of candidate segments, in which each element is a 5-tuple (*r*_*t*_, *z*_*t*_, *δ*_*t*_, *bp*_*t*_, *R*_*t*−1_), where *R*_*t*−1_ is the set of all segments extended to the current road segment at the time *t* − 1 according to the road network topology, and each element in *R*_*t*−1_ is a 4-tuple (*r*_*t*−1_, *z*_*t*−1_, *δ*_*t*−1_, *bp*_*t*−1_).
**createCR**(*p*_*t*_, *G*) An error region is set with *p*_*t*_ as the center and a certain length as the radius (The error radius can be the following: GPS accuracy + road network accuracy + road width. For example, the accuracy of civilian GPS is generally within 20 m. This paper implements the maximum value of 20 m. Therefore, given a road accuracy of 20 m, such as in GPS, and a two-lane road width of approximately 10 m, the error radius is 50 m.). Then, all the segments intersecting the region are selected as the candidate segments, and the candidate matching points formed by *p*_*t*_ that are projected onto the candidate segments are calculated to form a 5-tuple to construct the *CR*.
**extendCR**(*CR*_*t*−1_, *p*_*t*_, *G*) Starting from the road segment in *CR*_*t*−1_, the road segments are expanded according to the road network topology. Calculate the shortest distance from *p*_*t*_ to the extended segment. If the distance is greater than the error radius, the extended road segment will not be added to the set *CR*; otherwise, the extended road segment will be used as the candidate road segment at the time *t* to construct the *CR*, and all previous segments of each candidate road segment are added to the set *CR*_*t*−1_.
**insideJDD**(*p*) Determine if the point *p* is within the junction decision domain.

The initial matching algorithm (InitialMatching) is shown in Algorithm 1; the modified subsequent matching algorithm (SubsequentMatching) is shown in Algorithm 2, and the junction matching algorithm (JunctionMatching) is shown in Algorithm 3.

**Algorithm 1** Initial Matching

**Require**: *G*, *p*_*t*_, *MR*.

**Ensure**: The matching result at the time *t*.

1: *CR* ≔ createCR(*p*_*t*_, *G*)

2: **if** *CR* = ∅ **then**

3:  **return**

4: **end if**

5: *max*_*δ*_ ≔ 0

6: *mr* ≔ *null*

7: **for** *cr* ∈ *CR* **do**

8:  $cr.\delta ≔P(p_{t}|cr.r)≔\int_{-w}^{w}\frac{1}{\sqrt{2\pi }\sigma }exp\;\left(-0.5{\left(\frac{x-\|p_{t}-cr.z\|}{\sigma }\right)}^{2}\right)\;\text{d}x$

9:  **if** *cr*.*δ* > *max*_*δ*_ **then**

10:   *max*_*δ*_ ≔ *cr*.*δ*

11:   *mr* ≔ (*cr*.*r*, *cr*.*z*, *cr*.*δ*, *null*)

12:  **end if**

13: **end for**

14: **return** *mr*

**Algorithm 2** Subsequent Matching

**Require**: *G*, *p*_*t*_, *CR*_*t*−1_, *MR*.

**Ensure**: The matching result at the time *t*.

1: *CR* ≔ extendCR(*CR*_*t*−1_, *p*_*t*_, *G*)

2: *max*_*δ*_ ≔ 0

3: *mr* ≔ *null*

4: **for** *cr* ∈ *CR* **do**

5:  *m*_*δ*_ ≔ 0

6:  **for** *crr* ∈ *cr*.*R* **do**

7:   $P(p_{t}|cr.r)≔\int_{-w}^{w}\frac{1}{\sqrt{2\pi }\sigma }exp\;\left(-0.5{\left(\frac{x-\|p_{t}-cr.z\|}{\sigma }\right)}^{2}\right)\;\text{d}x$

8:   *P*(*cr*.*r*|*crr*.*r*) ≔ *β* × *exp* (−*β* × |‖*p*_*t*_ − *p*_*t*−1_‖ − ‖*cr*.*z* − *crr*.*z*‖|)

9:   *δ* ≔ *crr*.*δ* × *P*(*cr*.*r*|*crr*.*r*) × *P*(*p_t_*|*cr*.*r*)

10:   **if** *δ* > *m*_*δ*_ **then**

11:    *m*_*δ*_ ≔ *δ*

12:    *cr*.*δ* ≔ *δ*

13:    *cr*.*bp* ≔ *crr*

14:   **end if**

15:  **end for**

16:  **if** *cr*.*δ* > *max*_*δ*_ **then**

17:   *max*_*δ*_ ≔ *cr*.*δ*

18:   *mr* ≔ (*cr*.*r*, *cr*.*z*, *cr*.*δ*, *cr*.*bp*)

19:  **end if**

20: **end for**

21: *result* ≔ *mr*

22: *i* ≔ 1

23: **while** *mr* ≠ *null* && insideJDD(*p*_*t*−*i*_) **do**

24:  *mr* ≔ *mr*.*bp*

25:  **if** *mr* ≠ *null* **then**

26:   setMR(*MR*, *t* − *i*, *mr*)

27:   *i* ≔ *i* + 1

28:  **end if**

29: **end while**

30: **return** *result*

**Algorithm 3** Junction Matching

**Require**: *G*, *p*_*t*_, *CR*_*t*−1_, *MR*, *cp*–the intersection point.

**Ensure**: The matching result at the time *t*.

1: *CR* ≔ extendCR(*CR*_*t*−1_, *p*_*t*_, *G*)

2: *max*_*δ*_ ≔ 0

3: *mr* ≔ *null*

4: **for** *cr* ∈ *CR* **do**

5:  *m*_*δ*_ ≔ 0

6:  **for** *crr* ∈ *cr*.*R* **do**

7:   $P(p_{t}|cr.r)≔\int_{-w}^{w}\frac{1}{\sqrt{2\pi }\sigma }exp\;\left(-0.5{\left(\frac{x-\|p_{t}-cr.z\|}{\sigma }\right)}^{2}\right)\;\text{d}x$

8:   *P*(*cr*.*r*|*crr*.*r*) ≔ *β* × *exp* (−*β* × |‖*p*_*t*_ − *p*_*t*−1_‖ − ‖*cr*.*z* − *crr*.*z*‖|)

9:   *δ* ≔ *crr*.*δ* × *P*(*cr*.*r*|*crr*.*r*) × *P*(*p_t_*|*cr*.*r*)

10:   **if** *δ* > *m*_*δ*_ **then**

11:    *m*_*δ*_ ≔ *δ*

12:    *cr*.*δ* ≔ *δ*

13:    *cr*.*bp* ≔ *crr*

14:   **end if**

15:  **end for**

16:  **if** *cr*.*δ* > *max*_*δ*_ **then**

17:   *max*_*δ*_ ≔ *cr*.*δ*

18:   *mr* ≔ (*cr*.*r*, *cr*.*z*, *cr*.*δ*, *cr*.*bp*)

19:  **end if**

20: **end for**

21: **if** *mr* = *null* **then**

22:  **return** *null*

23: **else if** *mr*.*r* and getMR(*MR*, *t* − 1).*r* belong to the same road **then**

24:  **return** *mr*

25: **else**

26:  **return** *cp*

27: **end if**

## 4 Analysis of experimental results

### 4.1 Experimental method

The algorithm proposed in this paper is an improvement of the HMM-based matching algorithm; therefore, two representative HMM-based algorithms have been chosen for comparison. The main differences between the two algorithms are in the calculation method of the transition probability: the former is in accord with experience-based judgment, while the latter is the simplified form of the former and is a better fit for an HMM assumption. Applying the junction decision domain model of this paper to the algorithms of [13] and [14], respectively, two new algorithms that incorporate the decision domain of this paper can be formed: the improved algorithm of [13] and the improved algorithm of [14]. In addition, another method for defining the junction decision domain is mentioned above, that is, the method of [1], which does not consider the GPS accuracy, the road network accuracy, or the angle between the road segments, but instead completely calculates the range of the junction decision domain according to the width of the road segment. This paper also applies this junction decision domain definition method to improve the algorithms of [13] and [14] and names the improved new algorithms that incorporate the decision domain of [1]) as the improved algorithm of [13] and the improved algorithm of [14].

Thus far, this paper has built two groups of test algorithms. The first group is based on the algorithm of [13], including the algorithm of [13], the improved algorithm of [13] (the decision domain of this paper) and improved algorithm of [13] (the decision domain of [1]). The second group is based on the algorithm of [14], including the algorithm of [14], the improved algorithm of [14] (the decision domain of this paper) and the improved algorithm of [14] (the decision domain of [1]).

### 4.2 Experimental data

In this paper, two GPS data sets are tested. One is the data set (DS) made public in [13] (hereinafter referred to as “DS 1”), which is also used in [14]. The DS 1 was tested for approximately 80 km in Seattle, Washington, US, covering 7531 GPS points at a sampling rate of 1 Hz. Another DS is the actual road test data collected in this paper (hereinafter referred to as “DS 2”). The DS 2 was tested for approximately 30 km in Changchun, Jilin Province, China, covering 5330 GPS points at a sampling rate of 1 Hz.

To complete the map matching, road network data, in addition to GPS data, are also needed. For DS 1, [13] discloses road network data (hereinafter referred to as “road network data 1”), and for DS 2, the urban road network data of Changchun City (hereinafter referred to as “road network data 2”) are used. In both data sets, the road segments (or the road chains consisting of road segments) and the nodes are numbered to facilitate the establishment of the road network structure and the calculation of matching accuracy. In addition, both of the data sets contain no information on the road width, and only some of the roads in the road network data 2 are graded. Therefore, the road segment width can only be estimated by road grade.

### 4.3 Parameter determination

The parameters needed for the calculation of the observation probability and the state transition probability can be determined with the method in [13] or [14]. Especially for DS 1, the parameter values in the two references can be directly referred to.

The parameters inside the junction decision domain have three components: *σ*, *σ*′ and *l*(*c*). The first component *σ* shares the same symbol and meaning with the *σ* in Eq (3) and can be calculated with the method in [13] or [14]. *σ*′ can be equal to *σ*, as the road network can be assumed to be drawn with the data collected by a GPS device so that the two parameters have the same accuracy. *l*(*c*) is determined on a case-by-case basis. Thus, it is obvious that the parameter to focus on is *l*(*c*). To determine it, both road width and the road angle must be known. The road width, if available in the road network data, can be directly referred to; otherwise, it can only be assumed in accordance with the relevant road standard. For the road network data 1, it can be determined from the American road standard that the lane width is usually approximately 3.6 m. Considering that a road segment in a road network usually represents two lanes (one-way, two-lane or two-way, two-lane) and possible margins, the width of the road segment can be set to 8 m during the experiment. For the road network data 2, it can be determined from China Highway Engineering Technique Standard that the width of the main road of the city is generally approximately 3.5 m, so the width of the road can be set to 7 m. With the width of the road segment, *l*(*c*) can be calculated by Eqs (8) or (9). For example, in [13], *σ* is 4.07 m, and *σ*′ is equal to *σ*, which is also 4.07 m. Assuming that the width of the road segment is 8 m and the angle of the road segment at the intersection is 90°, the junction decision domain is: $4.07+4.07+0.5\times 8\times \sqrt{2}\approx 13.8$ m.

### 4.4 Result analysis

Because the algorithm proposed in this paper improves the overall matching performance by improving the matching performance in the junction decision domain, in the experimental process, the number of GPS points in the junction decision domain (*n*_*jdd*_) and the number of matching errors in the junction decision domain (*e*_*jdd*_) are specifically counted, and the matching accuracy rate in the junction decision domain ((*n*_*jdd*_ − *e*_*jdd*_)/*n*_*jdd*_) is calculated.

In this paper, two groups of algorithms are applied to test two data sets. The test results of DS 1 are shown in Table 1. The algorithm of [13] and the algorithm of [14] were performed twice. Taking the algorithm of [13] as an example, the first line of Table 1 displays the algorithm of [13] (the decision domain of this paper for statistics), indicating that the algorithm of [13] is used for matching, and the decision domain of this paper is only used to measure the junction matching performance of this algorithm, that is, to count the values of *e*_*jdd*_ and *n*_*jdd*_ in order to compare with the algorithm of the second line—the improved algorithm of [13] (the decision domain of this paper). Similarly, the third line displays the algorithm of [13] (the decision domain of [1] for statistics), indicating that the algorithm of [13] is used for matching, and the decision domain of [1] is only used to measure the junction matching performance of this algorithm to compare with the algorithm of the fourth line.

**Table 1:** Test result of DS 1.

Matching algorithm	Total matching error number	Matching accuracy	*n*_*jdd*_	*e*_*jdd*_	$\frac{\left(n_{jdd}-e_{jdd}\right)}{n_{jdd}}$
Algorithm of [13] (decision domain of this paper for statistics)	311	0.959	1902	222	0.883
Improved algorithm of [13] (decision domain of this paper)	96	0.987	1902	7	0.996
Algorithm of [13] (decision domain of [1] for statistics)	311	0.959	564	105	0.814
Improved algorithm of [13] (decision domain of [1])	232	0.969	564	26	0.954
Algorithm of [14] (decision domain of this paper for statistics)	531	0.929	1902	417	0.781
Improved algorithm of [14] (decision domain of this paper)	176	0.977	1902	62	0.967
Algorithm of [14] (decision domain of [1] for statistics)	531	0.929	564	225	0.601
Improved algorithm of [14] (decision domain of [1])	401	0.947	564	95	0.832

Through analysis of the statistical data in Table 1, the following conclusions can be made:

1. Applying the junction decision domain model in the HMM-based matching algorithm can effectively improve the matching performance. Regardless of whether the junction decision domain of this paper or the decision domain of [1] is used, the matching performance is improved. By comparing the data in rows 1, 2, 5 and 6 of Table 1, it can be concluded that the decision domain of this paper reduces the total number of matching errors by an average of 285 or 68.0%, and the number of matching errors in the junction decision domain is reduced by an average of 285 or 91.0%. Comparison of the data in rows 3, 4, 7 and 8 shows that the decision domain of [1] reduces the total number of matching errors by an average of 104.5 or 24.9%, and the number of matching errors in the junction decision domain is reduced by an average of 104.5 or 66.5%.
2. A more reasonable decision domain setting improves the matching performance more significantly. The junction decision domain of this paper provides a more obvious improvement of the matching performance than that of the decision domain of [1]. Regarding the two indicators of the total number of matching errors and the number of matching errors in the junction decision domain, the decision domain of this paper reduces them by 68.0% and 91.0% on average, respectively, while the decision domain of [1] reduces them on average by 24.9% and 66.5%. The decision domain of this paper has a significantly larger reduction in these two indicators than that of [1].
3. The improvement of the total matching performance is completely determined by the improvement of the matching performance in the junction decision domain. The amount of reduction in the total number of matching errors is the same as the reduction in *e*_*jdd*_. Taking the data in rows 1 and 2 of Table 1 as examples, the difference between the total matching error number and the difference of *e*_*jdd*_ is the same. That is, the matching algorithm based on the junction decision domain model does not affect the matching performance outside the junction decision domain, which is consistent with the original design of this algorithm. At the same time, this means that when comparing the performance of the algorithm, it is only necessary to compare the relevant statistics in the junction decision domain, namely, *n*_*jdd*_, *e*_*jdd*_ and (*n*_*jdd*_ − *e*_*jdd*_)/*n*_*jdd*_.

Data set 2 is tested again using two groups of algorithms, and the test results shown in Table 2 are obtained. Analysis of the statistical data in Table 2 reveals that the application of the junction decision domain model can indeed reduce the number of matching errors near the intersection, and the decision domain of this paper reduces the value significantly more than the decision domain of [1]. Comparing the data in rows 1, 2, 5 and 6 of Table 2, it can be concluded that the decision domain of this paper reduces the value of *e*_*jdd*_ by an average of 56 or 60.4%. Comparing the data in rows 3, 4, 7 and 8 shows that the decision domain of [1] reduces the value of *e*_*jdd*_ by an average of 18.5 or 18.6%.

**Table 2:** Test result of DS 2.

Matching algorithm	*n*_*jdd*_	*e*_*jdd*_	(*n*_*jdd*_ − *e*_*jdd*_)/*n*_*jdd*_
Algorithm of [13] (decision domain of this paper for statistics)	804	52	0.935
Improved algorithm of [13] (decision domain of this paper)	804	18	0.978
Algorithm of [13] (decision domain of [1] for statistics)	227	52	0.771
Improved algorithm of [13] (decision domain of [1])	227	43	0.811
Algorithm of [14] (decision domain of this paper for statistics)	799	141	0.824
Improved algorithm of [14] (decision domain of this paper)	799	63	0.921
Algorithm of [14] (decision domain of [1] for statistics)	227	141	0.379
Improved algorithm of [14] (decision domain of [1])	227	113	0.502

**Fig 9:**
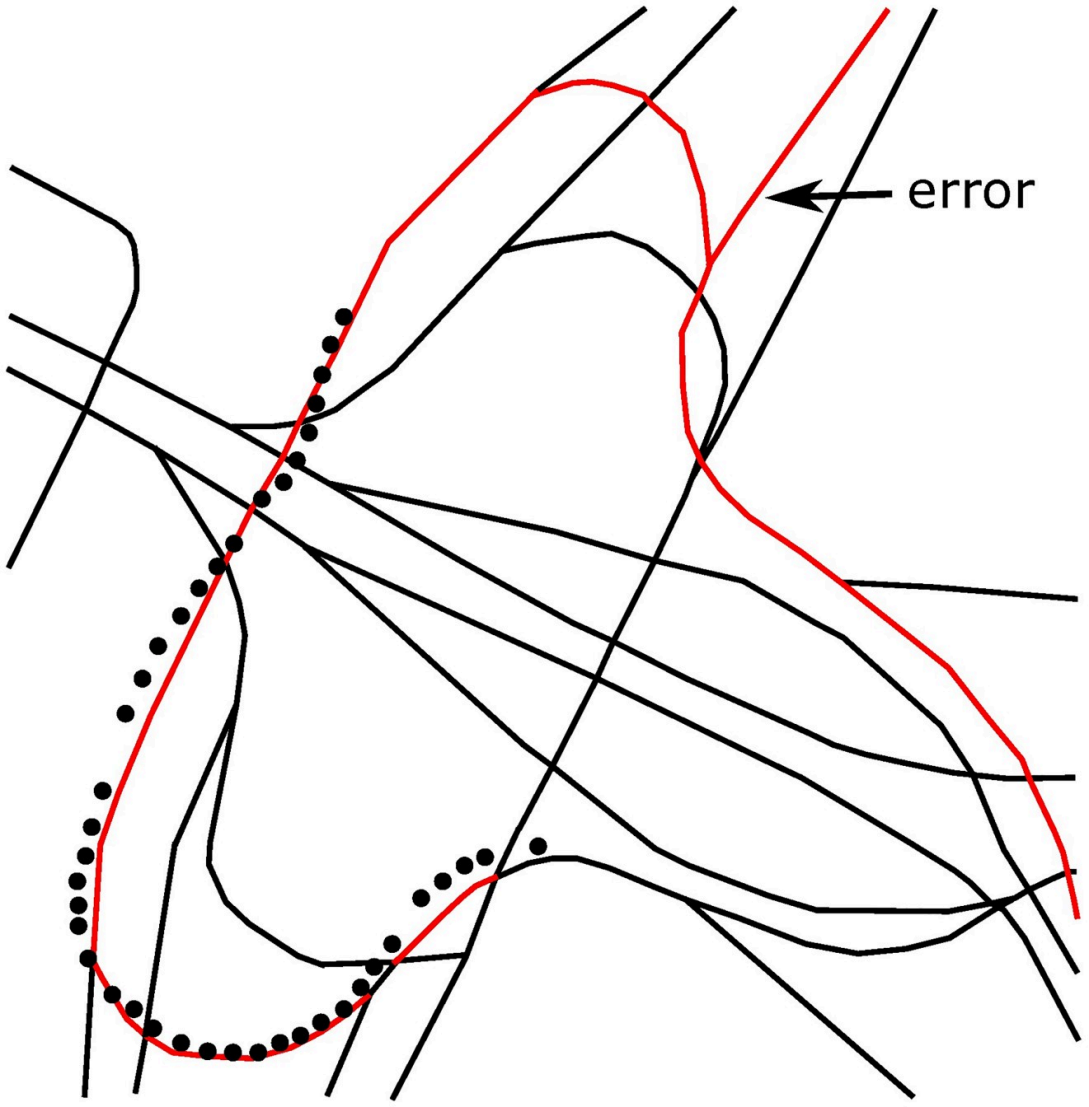
Part of the test chart of an overpass with the junction decision domain.

In addition, for an overpass with many junctions, only one mismatch has been caused by the algorithm proposed in this paper, as shown in Fig 9. Compared with the algorithm that made 5 mistakes, as shown in Fig 6, our algorithm also behaves very well in a complex road network.

## 5 Conclusion

Since an intersection is an area where a matching algorithm tends make mistakes and matching the error at an intersection can seriously influence the navigation system, this paper conducted an in-depth study of the matching problem at intersections and related solutions, presenting a decision domain model that is used to improve the HMM-based algorithm. The core strategy of the improved algorithm is delayed matching, that is, when GPS points are in the error-prone area (inside the junction decision domain), the matching result that is as correct as possible is output (with the intersection as the result). The experiment shows that this strategy is effective and that it can raise the matching accuracy at the intersection, thus improving the overall matching performance. The model of the junction decision domain can be applied not only to the HMM-based algorithm but also to other matching algorithms because delayed matching is a strategy that is unrelated to specific algorithms. This strategy can be applied to the MHT-based algorithm, the algorithm based on interval analysis and evidence theory, and other matching algorithms. The algorithm proposed in this paper only needs the inputs from an onboard GPS sensor and a digital map. Hence, compared with the matching algorithm integrated with the information of multiple sensors, this algorithm will provide a lower application cost.

## Supporting information

S1 File The description of the use of the DS 2 dataset. (DOCX)

S2 File The GPS data of the DS 2 dataset. (ZIP)

S3 File The road network data of the DS 2 dataset. (ZIP)
