# Supplementary material for: Map-matching algorithm based on the junction decision domain and the hidden Markov model
Source: PLoS One. 2019 May 13;14(5):e0216476. doi: 10.1371/journal.pone.0216476 (PMC6513071; doi:10.1371/journal.pone.0216476)
Supplement: S1 File — (DOCX) [file pone.0216476.s001.docx]

Data usage instructions

This document is a description of the use of the DS 2 dataset, which consists of two parts: GPS data and road network data. You can download GPS data from <http://www.custdev.club/ds2/gps.zip> and download road network data from <http://www.custdev.club/ds2/vnc.zip>. Road network data is MySQL database file, please use the table v_roadnet after importing the database. The file format of GPS data is: 8 bytes of time, 8 bytes of longitude (double type), 2 bytes of longitude identification, 8 bytes of latitude (double type), 2 bytes of latitude identification, 8 bytes of speed (double type) and 8 bytes of direction (double type).
